# Recombinant transgelin‐like protein 1 from *Mytilus* shell induces formation of CaCO_3_ polymorphic crystals *in vitro*

**DOI:** 10.1002/2211-5463.12972

**Published:** 2020-09-21

**Authors:** Yuting Jiang, Qi Sun, Meihua Fan, Jianyu He, Xiaolin Zhang, Huanzhi Xu, Zhi Liao

**Affiliations:** ^1^ Laboratory of Marine Biology Protein Engineering Marine Science and Technical College Zhejiang Ocean University Zhoushan City China; ^2^ Department of Biology University of Pisa CoNISMa Italy

**Keywords:** biomineralization, CH domain, *Mytilus coruscus*, shell matrix proteins, transgelin‐like protein

## Abstract

Transgelin is an actin cross‐linking/gelling protein of the calponin family, which is associated with actin stress fibres, cell motility, adhesion and the maintenance of cell morphology. Transgelin‐like proteins (TLPs) have also been identified as shell matrix proteins (SMPs) in several mollusc species; however, the functions of TLPs in biomineralization remain unknown. Transgelin‐like protein 1 (TLP‐1) was previously identified from the shell of *Mytilus coruscus* as a novel 19 kDa SMP with a calponin homology (CH) domain. To understand the role of TLP‐1 in shell formation, the expression level and localization of the TLP‐1 gene in biomineralization‐related tissues were determined in this study. Furthermore, recombinant TLP‐1 was expressed in a prokaryotic expression system with codon optimization, and an anti‐rTLP‐1 antibody was prepared based on the expressed recombinant TLP‐1 (rTLP‐1) protein. *In vitro*, rTLP‐1 induced the formation of CaCO_3_ polymorphic crystals with distinct morphologies and inhibited crystallization rate and crystal interactions. Immunohistochemical, immunofluorescence, and pull‐down analyses using the anti‐rTLP‐1 antibody revealed the specific locations of TLP‐1 in biomineralization‐related tissues and shell myostracum layer, and suggested the existence of a possible TLP‐1 interaction network in the shell matrix. Our results are beneficial for understanding the functions of TLP‐1, particularly through its CH domain, during shell mineralization.

## Graphical abstract

Transgelin‐like protein 1 (TLP‐1) was identified from the shell of *Mytilus coruscus* as a novel 19 kDa shell matrix protein with a calponin homology domain. Localization of TLP‐1 in biomineralization‐related tissues was determined. The effects of recombinant TLP‐1 on calcium carbonate crystallization were studied to determine its possible function in shell mineralization.

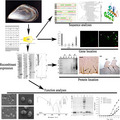

**Abbreviations**
AA: amino acid
AIS: acid‐insoluble
AMS: adductor muscle scar
AS: acid‐soluble
BLI: biolayer interferometry
BSA: bull serum albumin
CH: calponin homology
CLP: collagen‐like protein
DAB: diaminobenzidine
DAPI: 4′, 6‐diamidino‐2‐phenylindole
FDR: false discovery rate
FTIR: fourier transform infrared spectroscopy
HPLC: high‐performance liquid chromatography
LC‐MS/MS: liquid chromatography tandem mass spectrometry
MW: molecular weight
PBS: phosphate buffer saline
PCR: polymerase chain reaction
SEM: scanning electron microscopy
SMP: shell matrix protein
TLP: transgelin‐like protein
WLP: whirlin‐like protein

For bivalves, biomineralization is a process intimately associated with shell matrix proteins (SMPs) secreted by the mantle. These SMPs are characterized by their ability to control the formation of bivalve shells with different morphologies and crystal polymorphs [1, 2]. Bivalve shells are generally composed of approximately 95% calcium carbonate (aragonite and calcite) and 5% organic matrices consisting principally of SMPs [3]. During shell formation, CaCO_3_ crystals are deposited as calcite and aragonite, which form polymorphic nanostructures/layers with different morphologies, and finally assembled into calcified shells [4, 5]. Structural and functional studies of SMPs will advance the understanding of the shell formation process and the molecular mechanism of the excellent mechanical properties of these shells.

*Mytilus coruscus* is an edible mussel with important economic and ecological value in the East China Sea. Three layers (nacre, fibrous prism and myostracum) were observed by scanning electron microscopy (SEM), and more than 60 SMPs were previously identified in the shell of *M. coruscus* through transcriptome‐proteome strategies [6]. Among the shell proteome of *M. coruscus*, three novel SMPs with transgelin‐like structures were found, one of which, transgelin‐like protein 1 (TLP‐1, GenBank Accession: MT240932), was identified as a myostracum‐related SMP [6]. In *bivalves*, the myostracum is located in the attachment of the adductor muscle, commonly called the adductor muscle scar (AMS), to the umbo of each valve [6, 7]. The AMS is the surface of the myocardial support for the attachment of the adductor muscle and controls shell closure. However, studies on myostracum‐related SMPs are rare.

Transgelin and smooth muscle 22 alpha [8] are shape‐change sensitive actin cross‐linking/gelling proteins of the calponin family [9]. Transgelin is ubiquitous in vascular and visceral smooth muscle cells and is associated with actin stress fibres participating in cell motility, adhesion and the maintenance of cell morphology [10, 11, 12, 13]. As a member of the calponin family, transgelin contains a sequence motif of approximately 100 amino acids termed the ‘calponin homology (CH) domain’ [14]. The CH domain has been suggested to enable actin binding to a variety of cytoskeletal and signalling molecules [15]. In bivalves, TLPs have been identified in the shell matrices of several species, such as *M. coruscus* [6], *Mytilus galloprovincialis* [16], *Crassostrea gigas* [17] and *Perna viridis* [18], indicating possible roles for transgelin in shell formation. However, the relationship between transgelin and biomineralization has not been described to date. Interestingly, it has been reported that calponin, with a CH domain similar to transgelin, acts as a negative factor during the process of bone mineralization in vertebrates, revealing a biomineralization‐related function of calponin in vertebrates [19, 20]. In addition, the biomineralization‐related functions of calponin were reported recently in bivalve *Chlamys farreri* [21]. Considering that the both calponin and transgelin have a CH domain, we speculate that TLP might also be associated with shell formation in *bivalves*. In addition, because TLP‐1 does not contain calponin repeat domains [21], having only a single CH domain, TLP‐1 is a molecular model useful for exploring the function of the CH domain in biomineralization. Therefore, TLP‐1 from *M. coruscus* was recombinantly expressed using a *pET28*α*/Escherichia coli* system, and the effects of recombinant TLP‐1 on calcium carbonate crystallization were studied to reveal a possible function of TLP‐1 in shell mineralization. Additionally, the localization of TLP‐1 in biomineralization‐related tissues, such as the mantle, adductor muscle and shell inner surface, was detected. A possible interaction network of TLP‐1 in shell matrices was also analysed using the pull‐down technique. Our findings are beneficial for understanding the functions of TLP‐1, particularly its CH domain, during shell mineralization.

## Results

### Features of the TLP‐1 sequence

**Fig. 1:**
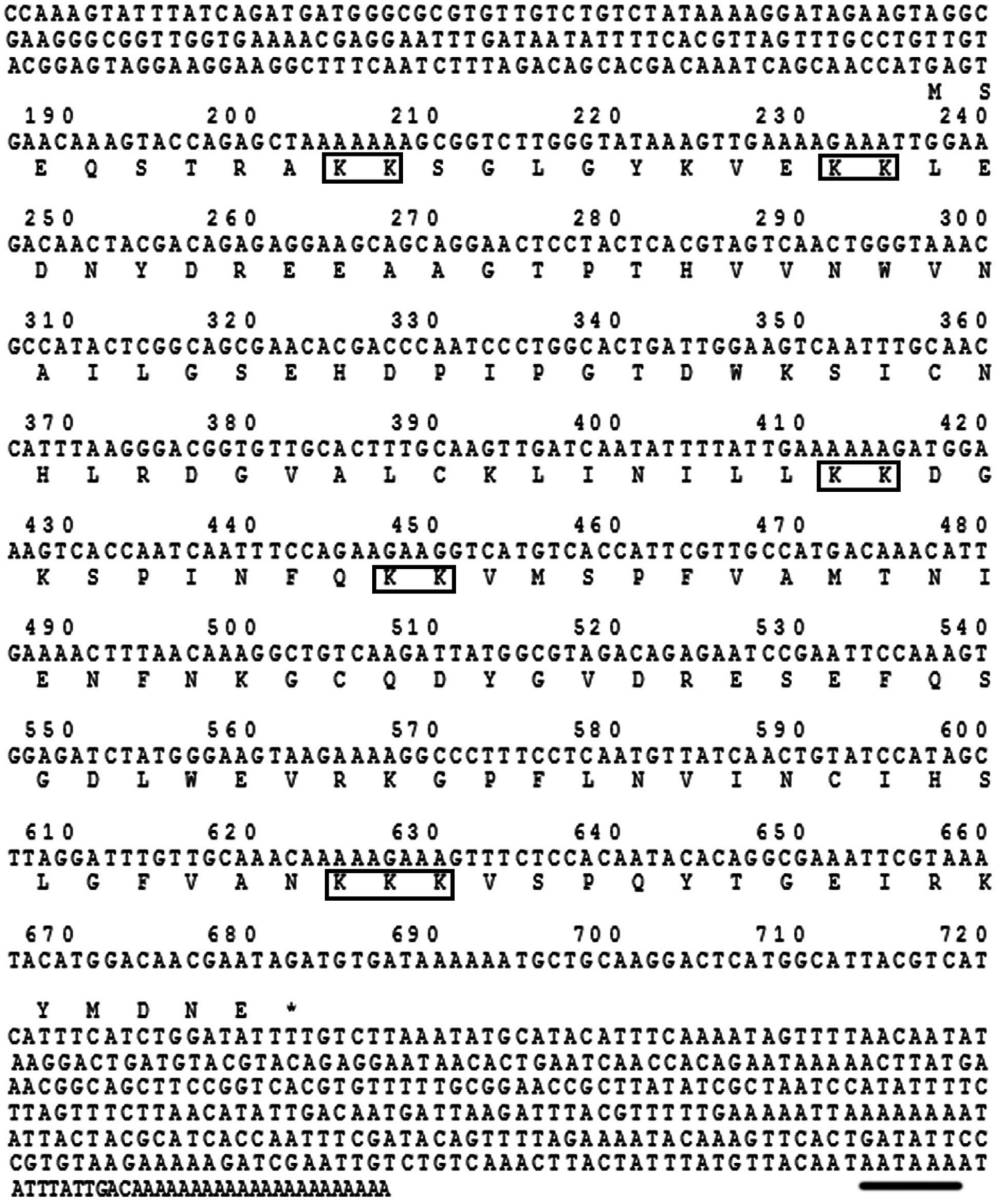
Alignment of the cDNA sequence of TLP‐1 with the deduced amino acid sequence. The termination codon was denoted by an *, the dipeptides of ‘‐KK‐’ were denoted by frames, and the tailing signal is underlined.

**Fig. 2:**
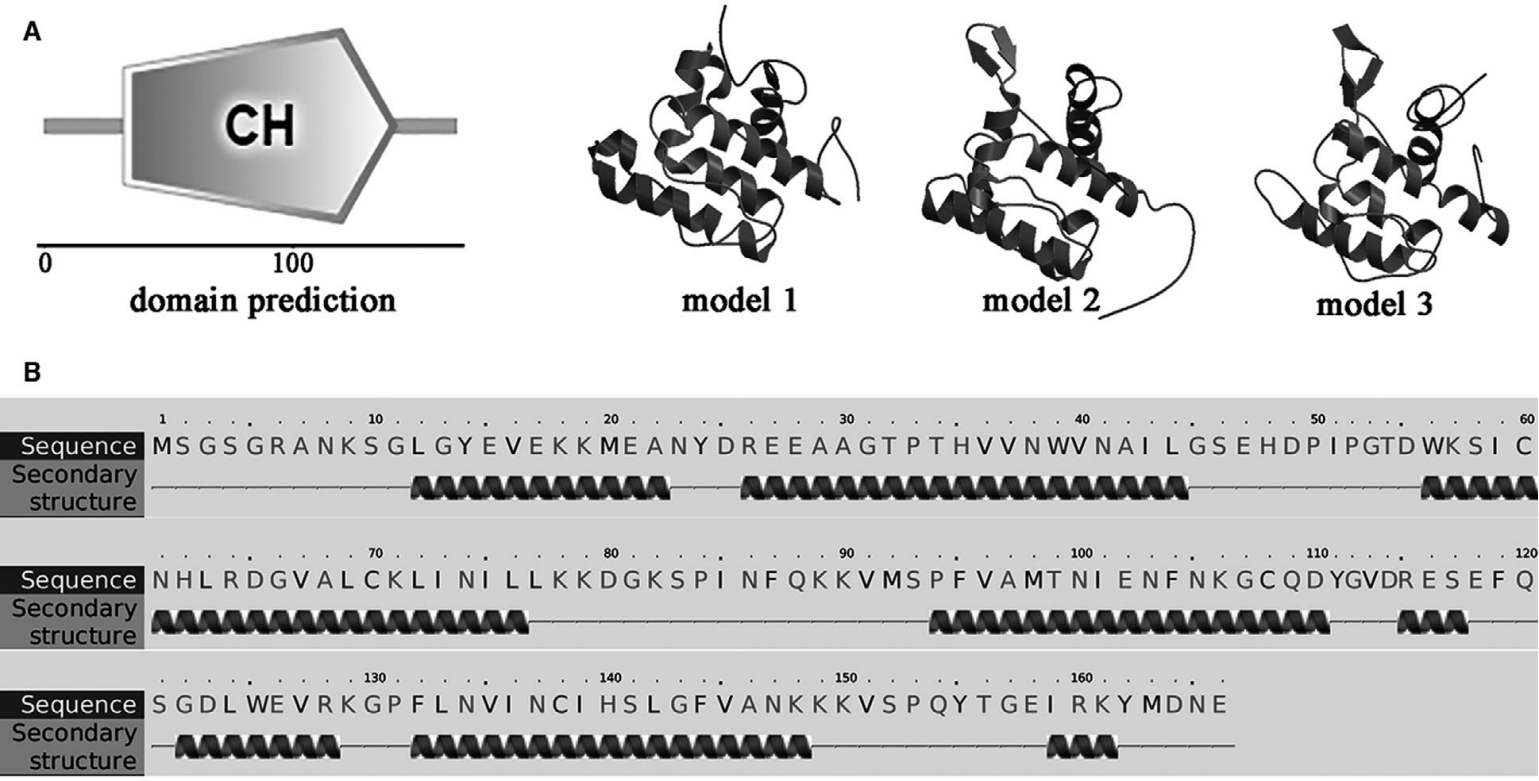
The structural features of TLP‐1. (A) domain prediction of TLP‐1 by SMART and one CH domain was presented. The spatial structure of TLP‐1 predicted by SWISS‐MODEL. Model 1–3 represent the predicted structures with highest score using 1wym.1.A, 1wyn.1.A and 1wyp.1.A as templates, respectively. (B) the secondary structure of TLP‐1 predicted by Phyre. The regions adopting putative α‐helix are represented as spirals.

**Fig. 3:**
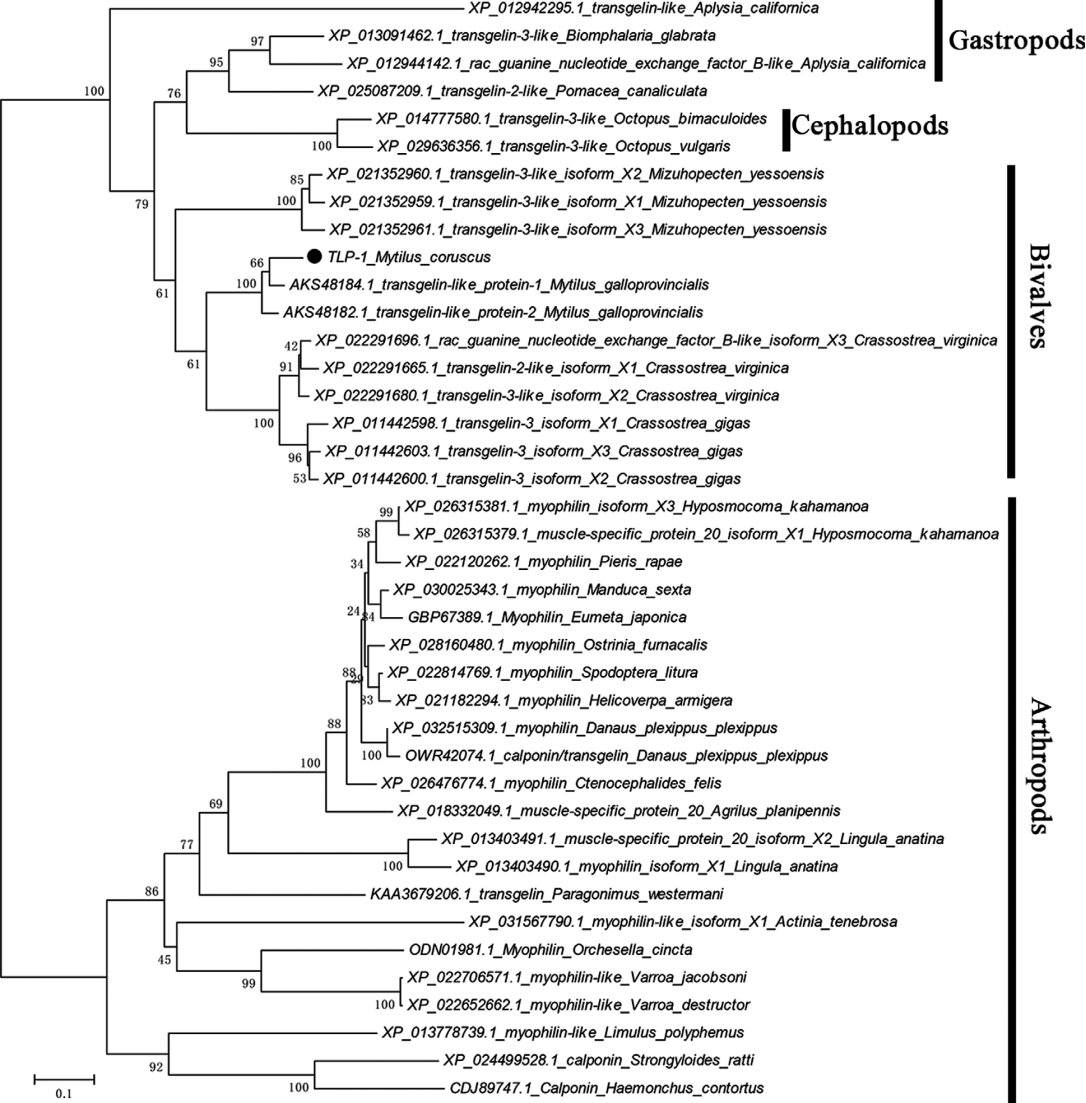
Phylogenetic analysis of TLP‐1. The phylogenetic tree was constructed using the Neighbour‐joining method with Poisson model (1000 bootstrap replications) in mega software 7.0. Homologous protein included in construction of phylogenetic tree was all retrieved from NCBI nr database with high score using blast. The rTLP‐1 was denoted by a circle. The information of sequences for construction of the tree are listed in Table S1.

Bioinformatics analysis revealed that the full length of the native TLP‐1 cDNA sequence is 112 bp and encodes a 167 amino acid (AA) precursor with a theoretical molecular weight (MW) of 18.89 kDa and an isoelectric point (*pI*) of 8.28 (Fig. 1). No signal peptide but one CH domain (residues 33–143) was detected in the TLP‐1 precursor (Fig. 2A). The secondary structure of TLP‐1 is primarily composed (59%) of α‐helices (Fig. 2B), forming a predicted tertiary structure with characteristics similar to the conformation reported for human transgelin‐2 (PDB ID: 1WYM) and mouse transgelin (PDB ID: 1UJO), which also contain abundant α‐helices. A protein blast search revealed that TLP‐1 shares high sequence identity (32–93%) with transgelin, calponin and myophilin in other invertebrates (Fig. S1 and Table S1). To examine this robust phylogenetic marker, a total of 39 representative homologous protein sequences were selected to construct a phylogenetic tree and perform a cluster analysis of orthologous genes. In the phylogenetic tree, two conspicuous branches were generated that included transgelin from gastropods/cephalopods/bivalves and myophilin/calponin from arthropods (Fig. 3 and Table S1). TLP‐1 was located in the *bivalve* branch and grouped closely with *Mytilus* (Fig. 3).

### Tissue expression and *in situ* hybridization

**Fig. 4:**
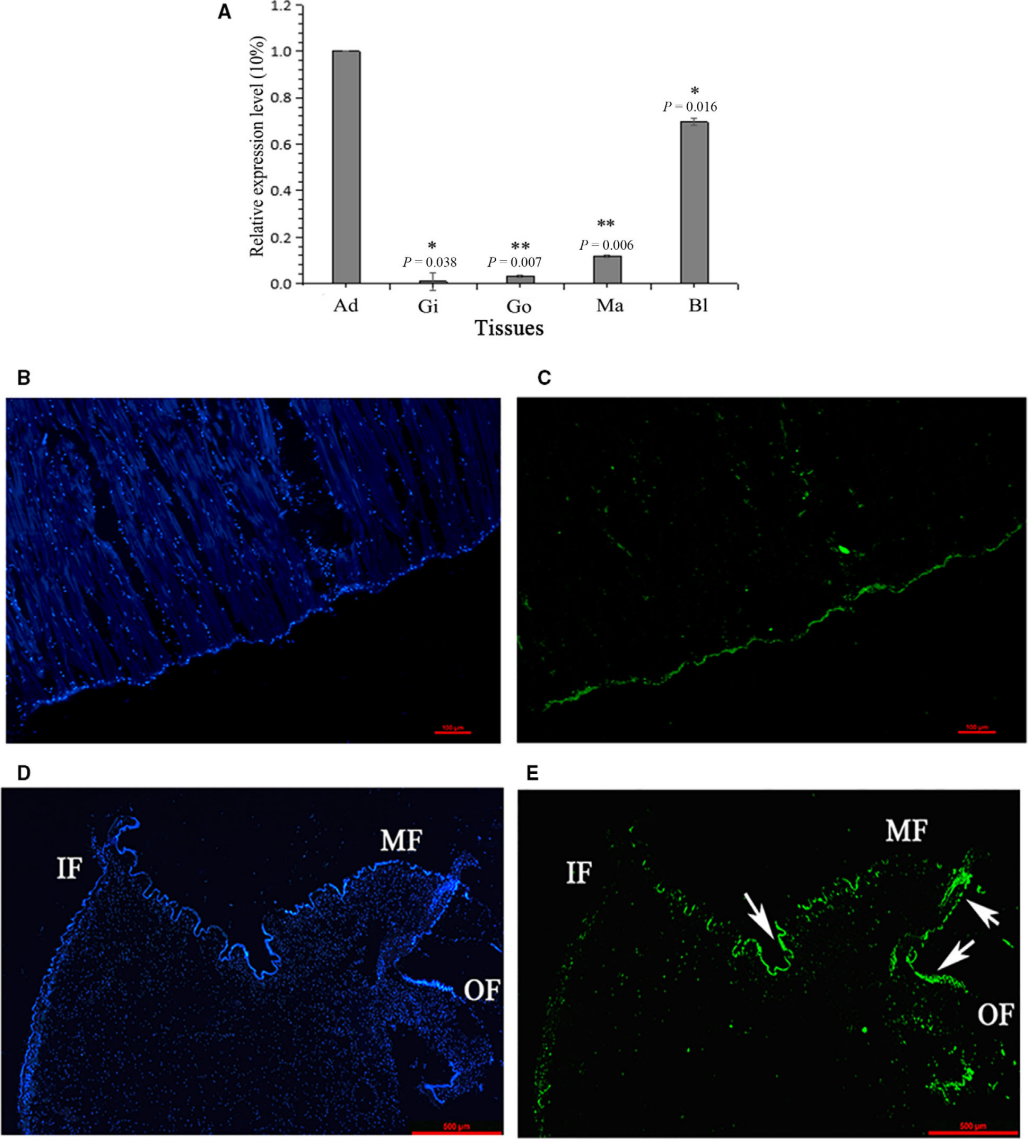
Tissue‐specific expression (A) and *in situ* hybridization (B–E) of TLP‐1. (A) Ad, adductor muscle; Gi, gill; Go, gonad; Ma, mantle; Bl, blood cell. Values for qPCR are means ± SD of three replicates. The statistical analysis of differences was performed by spss 16.0 (IBM SPSS Predictive Analytics Community, Armonk, New York, USA) with one‐way ANOVA followed by Tukey's multiple range test. *P < 0.05; **P < 0.01. (B) control group for adductor muscle; (C) expression of TLP‐1 (green colour) in adductor muscle; (D) control group for mantle; (E) expression of TLP‐1 (green colour) in mantle. The scale bar, 500 µm.

The tissue‐specific expression of TLP‐1 was investigated by fluorescence and quantitative real‐time PCR (qRT‐PCR). The mRNA transcripts of TLP‐1 were detected in all tested tissues. The highest expression level of TLP‐1 was observed in the adductor muscle, followed by the blood cell, mantle, gonad and gill (Fig. 4A). Using FAM‐labelled TLP‐1‐specific probes, strong signals were detected at the edge of the middle fold of the mantle and the bottom of the adductor muscle, close to the shell inner surface (Fig. 4B–E).

### Recombinant expression and the biomineralization‐related functions of rTLP‐1

**Fig. 5:**
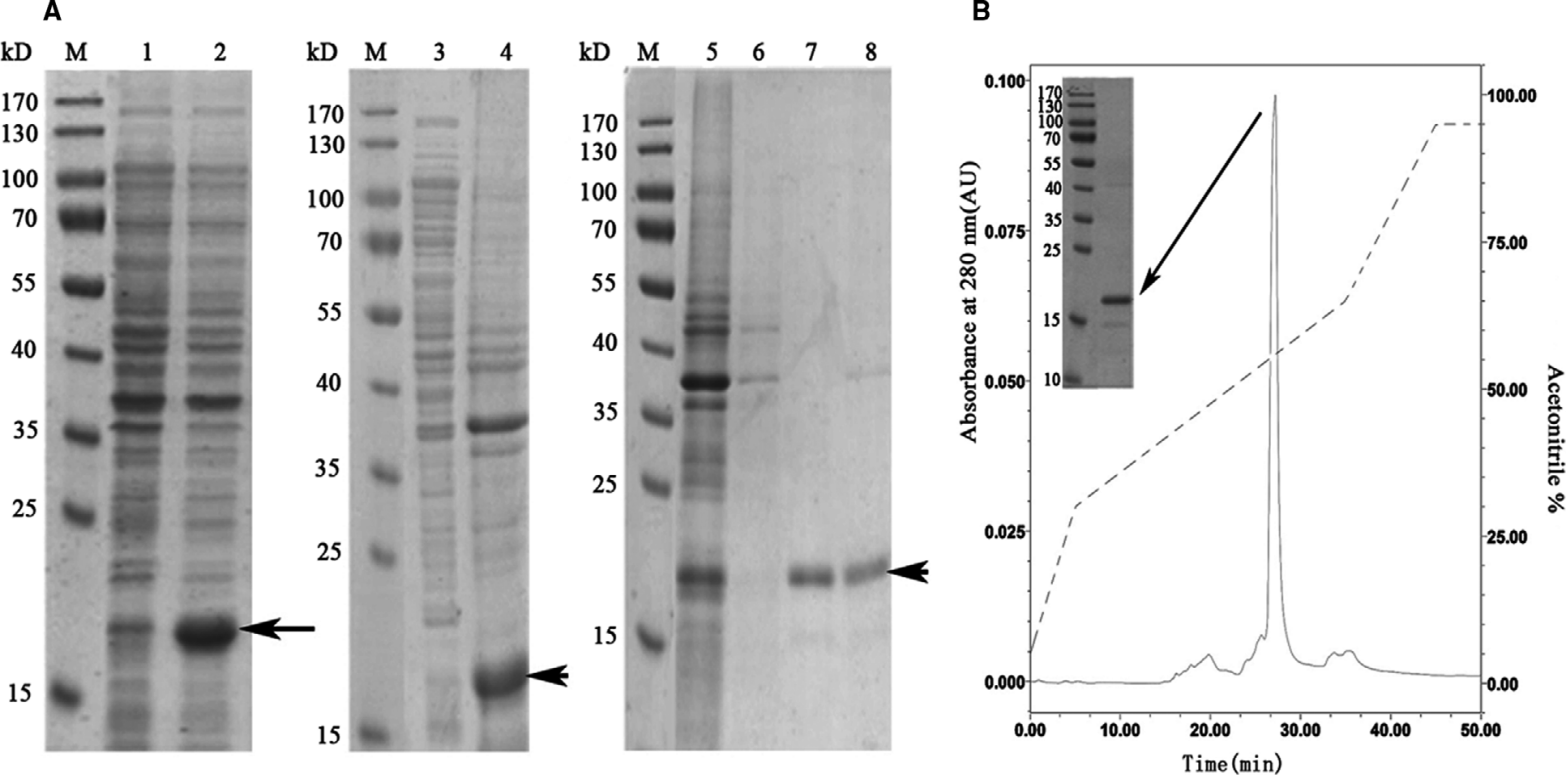
Recombinant expression and purification of TLP‐1. (A) SDS/PAGE analysis of recombinant expressed rTLP‐1. Lane M, Protein marker; Lane 1, negative control without IPTG induction; Lane 2, recombinant expression of TLP‐1 with induction of IPTG; Lane 3, the supernatant of the cell lysate; Lane 4, debris of the cell lysate; lane 5, penetrated sample from Ni‐NTA column; lane 6: eluted sample from Ni‐NTA column with 30 mm imidazole. Lane 7, eluted sample with 100 mm imidazole. Lane 8, eluted sample with 300 mm imidazole. The protein band with ~ 19 kD (indicated by arrows) corresponds to the rTLP‐1. (B) HPLC purification of rTLP‐1 after refolding. Elution profile of recombinant expressed rTLP‐1 on RP‐HPLC with a C8 column. Arrow denotes the target elution peak eluted at acetonitrile of 54%. The eluted sample was further tested by SDS/PAGE and the target protein band is denoted by an arrow.

In this work, the cDNA of *M. coruscus* TLP‐1 was codon optimized and synthesized and then inserted into an expression vector (pET 28α) containing a His_6_‐tag and an IPTG‐inducible T7 promoter to express recombinant TLP‐1 (rTLP‐1). Using this recombinant vector, we were able to successfully overexpress rTLP‐1 in an *E. coli* strain. The SDS/PAGE results revealed that rTLP‐1 with an expected theoretical MW of ~ 19 kDa was found in the insoluble fraction of the cell lysate (Fig. 5A). rTLP‐1 was then isolated by Ni column, refolded in buffer and purified using HPLC (Fig. 5B).

**Fig. 6:**
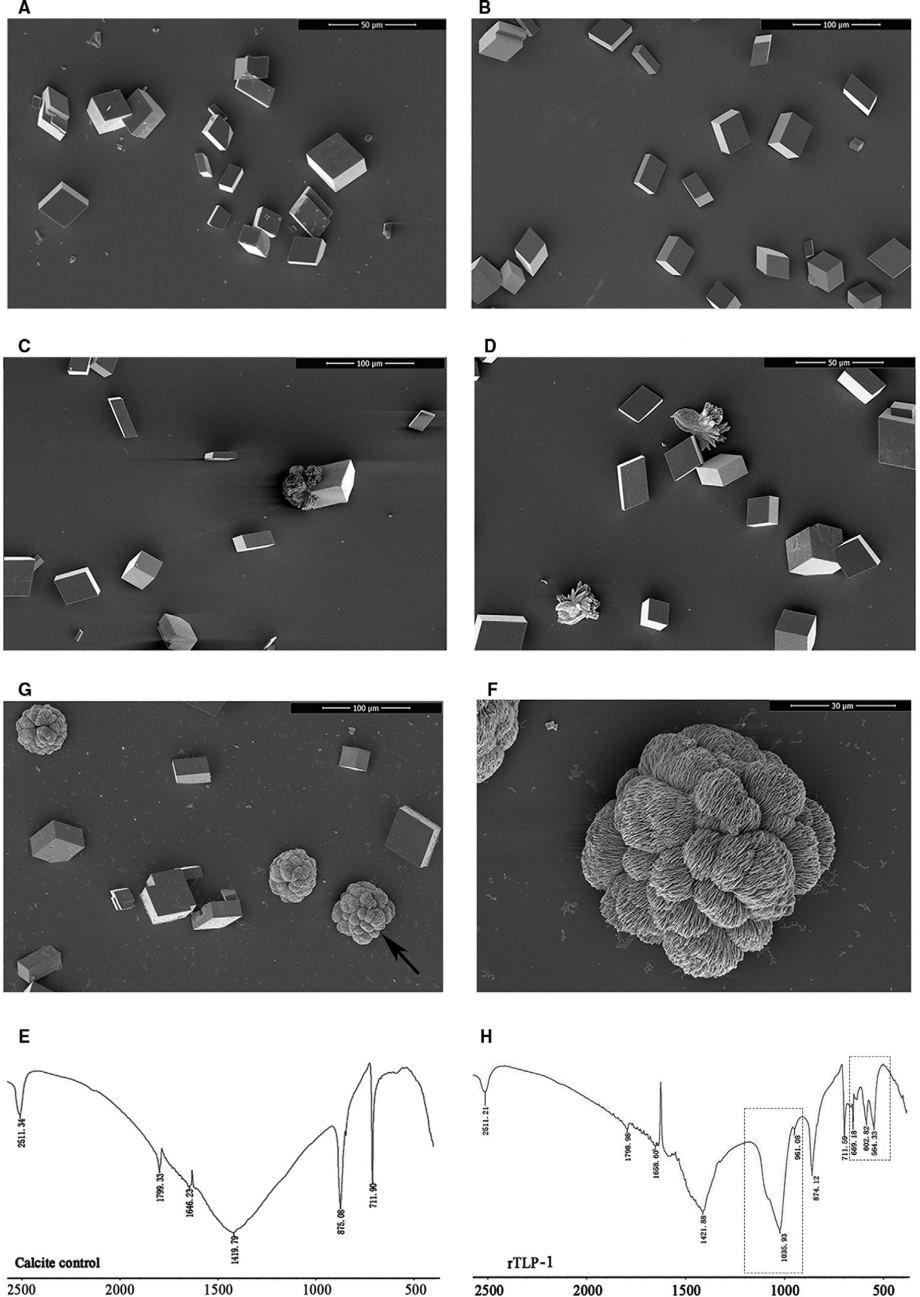
SEM images and FTIR spectra of *in vitro* calcite crystallization in the presence of rTLP‐1 at increasing concentrations. (A) calcite crystals grown without protein induction; (B) crystals grown with 50 μg·mL^−1^ BSA; (C) crystals grown with 10 μg·mL^−1^ rTLP‐1; (D) crystals grown with 30 μg·mL^−1^ rTLP‐1; (E) crystals grown with 50 μg·mL^−1^ rTLP‐1. (F) enlarged image of E; (G) FTIR spectrum of control calcite crystals; (H) FTIR spectrum of calcite crystals induced by 50 μg·mL^−1^ rTLP‐1. The frames indicate the transformed region by 50 μg·mL^−1^ rTLP‐1 induction. Scale bars, 50 μm for A and D; 100 μm for B, C and E; 30 μm for F.

**Fig. 7:**
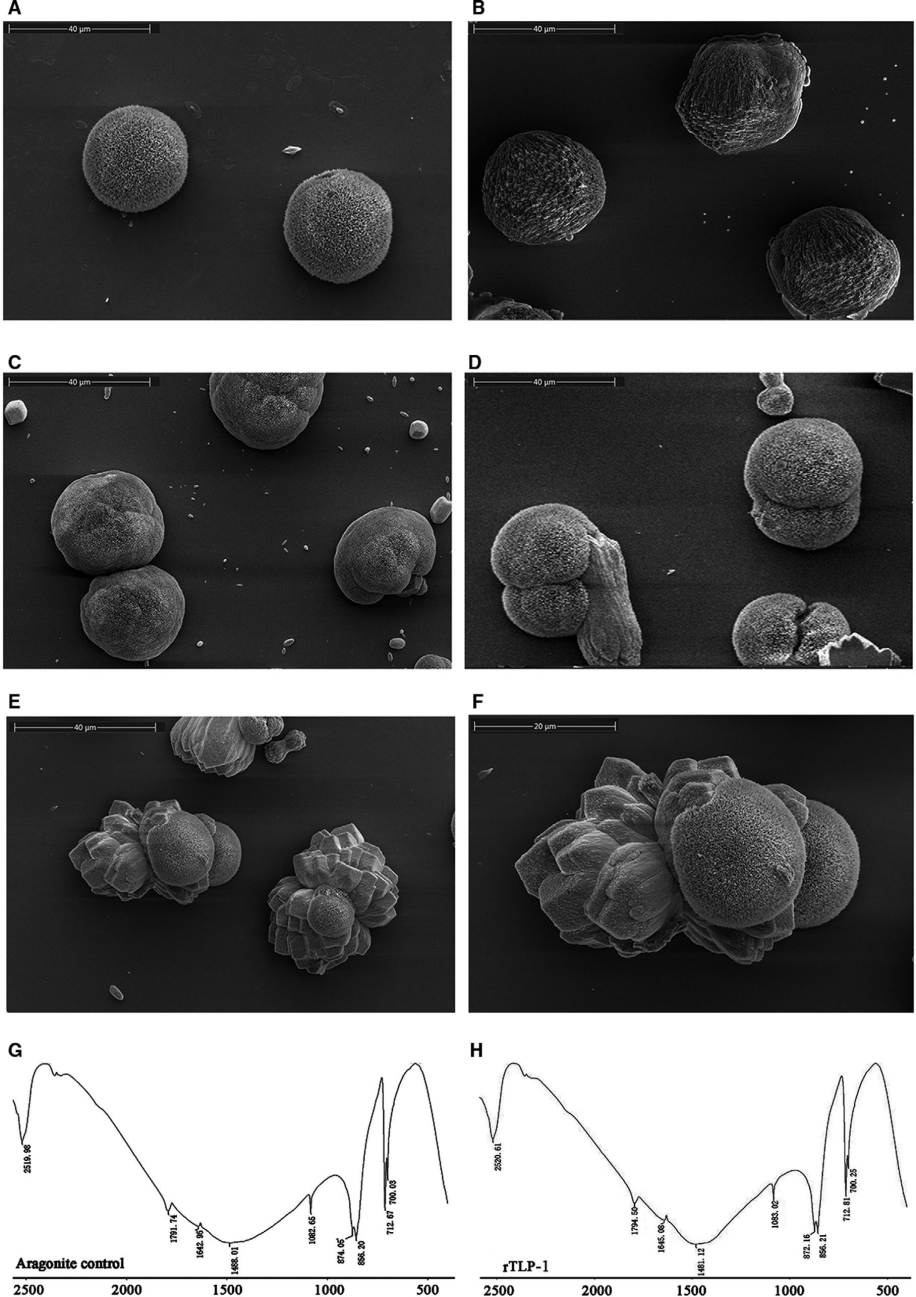
SEM images and FTIR spectra of *in vitro* aragonite crystallization in the presence of rTLP‐1 at increasing concentrations. (A) aragonite crystals grown without protein induction; (B) aragonite crystals grown with 50 μg·mL^−1^ BSA; (C) aragonite crystals grown with 10 μg·mL^−1^ rTLP‐1; (D) aragonite crystals grown with 30 μg·mL^−1^ rTLP‐1; (E) aragonite crystals grown with 50 μg·mL^−1^ rTLP‐1; (F) enlarged image of E; (G) FTIR spectrum of control aragonite crystals; (H) FTIR spectrum of aragonite crystals induced by 50 μg·mL^−1^ rTLP‐1. Scale bars, 40 μm for A–E; 20 μm for F.

Using *in vitro* CaCO_3_ crystallization assays, the effects of rTLP‐1 on the morphology of both calcite and aragonite crystals (Figs 6 and 7) were observed. Compared to the morphologies of the control samples (Fig. 6A,B), no significant morphological change was found in the calcite crystals when rTLP‐1 was added at a low concentration (10 μg·mL^−1^) (Fig. 6C). Few calcite crystals presented morphological changes after treatment with rTLP‐1 at high concentrations, and crystals were observed with structures that ranged from a radial (30 μg·mL^−1^, Fig. 6D) to a hydrangea‐like morphology (50 μg·mL^−1^, Fig. 6E,F). The polymorphs of the crystals formed upon rTLP‐1 treatment were characterized by FTIR. As shown in Fig. 6, the control calcite samples presented with calcite‐specific wavenumbers of 875 cm^−1^ and a single wavenumber of 711 cm^−1^ (Fig. 6G). The crystals formed by rTLP‐1 induction showed extra wavenumbers in the 1035 cm^−1^ and 564–669 cm^−1^ regions, with the 1035 cm^−1^ peak close to the aragonite‐specific peaks (1082 cm^−1^) (Fig. 6H), suggesting that rTLP‐1 induced the formation of aragonite.

For the aragonite crystals, rTLP‐1 showed significant effects on the crystal morphologies even at low concentrations. As shown in Fig. 7, natural globular aragonite crystals (Fig. 7A,B) changed with increasing concentrations of rTLP‐1, exhibiting a shrinking surface (10 μg·mL^−1^, Fig. 7C), a splice‐induced sphere morphology (30 μg·mL^−1^, Fig. 7D) and an irregular petal structure (50 μg·mL^−1^, Fig. 7E,F). No change in the polymorphs was detected for the rTLP‐1‐induced aragonite (Fig. 7G,H), indicating that rTLP‐1 had no effect on the polymorphs of the aragonite crystals.

**Fig. 8:**
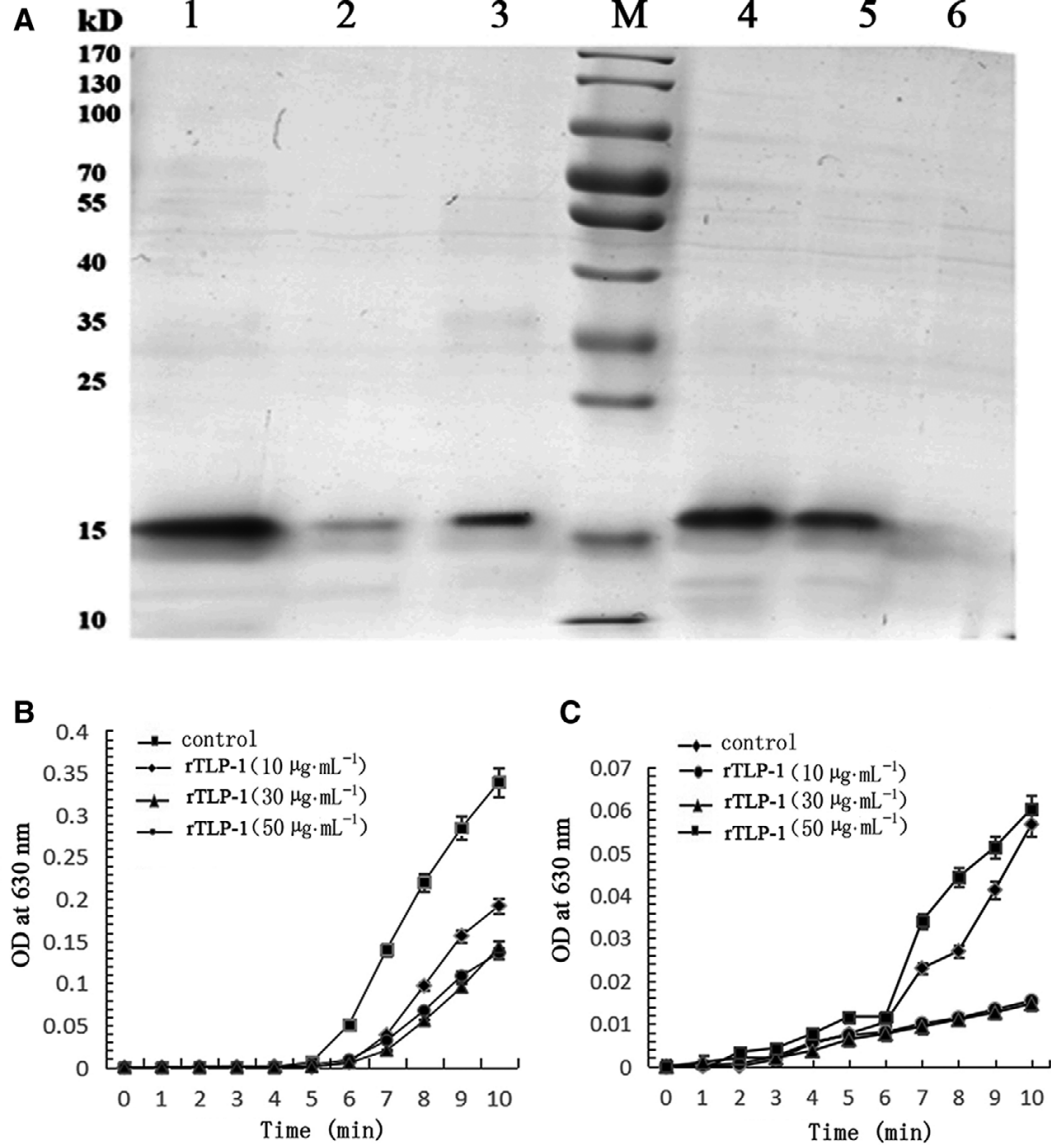
Binding ability of rTLP‐1 to calcite and aragonite (A), and the crystallization rate inhibition of rTLP‐1 in calcite (B) and aragonite (C), respectively. Lane M, protein marker; Lane 1 and Lane 4, pure rTLP‐1; Lane 2, supernatant of the solution after that the rTLP‐1 was precipitated by calcite crystals; Lane 3, the rTLP‐1 released from the precipitates of calcite crystals; Lane 5, supernatant of the solution after that the rTLP‐1 was precipitated by aragonite crystals; Lane 6, the rTLP‐1 released from the precipitates of aragonite crystals The data represent mean ± SD (*n* = 3).

A possible interaction between rTLP‐1 and CaCO_3_ crystals was detected by SDS/PAGE. As shown in Fig. 8A, lanes 1, 2 and 3 contained the pure rTLP‐1 solution, the supernatant from the rTLP‐1 solution after precipitation by CaCO_3_ crystals, and the rTLP‐1 released from the precipitated CaCO_3_ crystals, respectively. For the calcite crystals, the protein band of rTLP‐1 in lane 2 was much less intense than that in lane 1, revealing the binding of rTLP‐1 with the crystals, a finding that was confirmed by the reappearance of the protein band in lane 3. For the aragonite crystals, lane 4 presents the pure rTLP‐1 solution, and the protein band of rTLP‐1 was also observed in lane 5. Moreover, no protein bands were observed in lane 6 (Fig. 8A), suggesting that the binding of rTLP‐1 with aragonite crystals was much weaker than that with the calcite crystals.

The crystallization rate of CaCO_3_ was measured by the absorbance at 630 nm. As shown in Fig. 8B, rTLP‐1 significantly inhibited the crystallization rate of calcite crystals in a dose‐dependent manner, and the highest OD630 values (50 μg·mL^−1^ of rTLP‐1) were no higher than 0.2, which differed from that of the control group, at 0.35. For the crystallization rate of aragonite, rTLP‐1 showed slight promoting effects at 50 μg·mL^−1^, with the highest value of 0.06, and the value was 0.05 for the control group, and inhibiting effects at low concentrations (10 and 30 μg·mL^−1^) (Fig. 8C).

### Localization of TLP‐1 in shell matrices and tissues

**Fig. 9:**
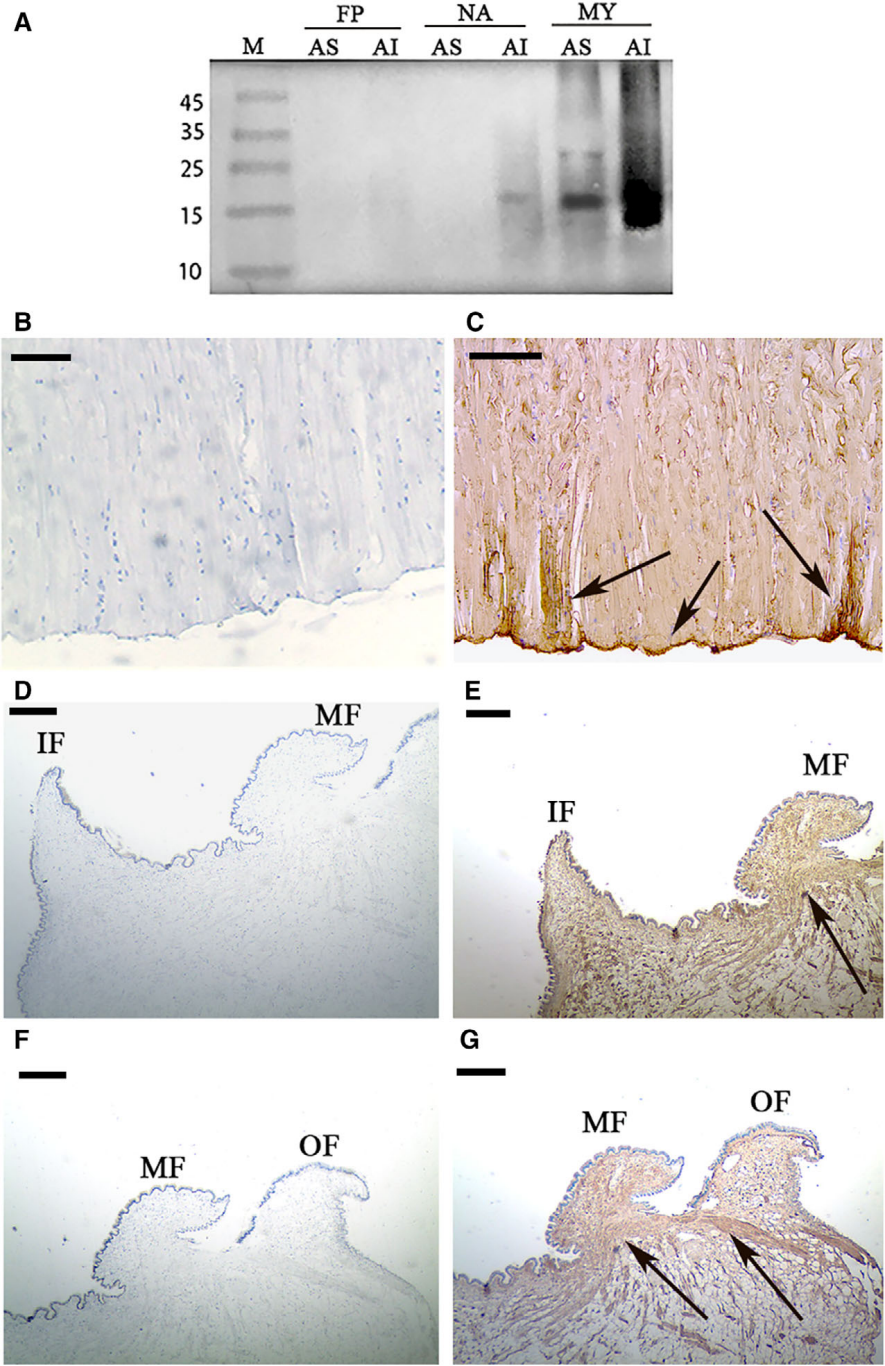
Western blot and immunohistochemistry analysis of TLP‐1. (A) Western blot by anti‐rTLP‐1 antibody in shell matrices. FP. fibrous prism; NA, nacre; MY, myostracum; AS, acid soluble; AI, acid insoluble; (B) immunohistochemistry analysis with the control group of adductor muscle performed using only second antibody showed no significant signals; (C) detection of TLP‐1 in the adductor muscle and the positive signals are indicated by arrows; (D) the control group of mantle, showing the inner and the middle fold; (E) detection of TLP‐1 in the mantle and the positive signals are indicated by an arrow; (F) the control group of mantle, showing the middle and the outer fold; (G) detection of TLP‐1 in the mantle and the positive signals are indicated by arrows. The scale bar, 50 µm.

In this study, a polyclonal anti‐rTLP‐1 antibody was prepared, and the localization of TLP‐1 in the matrices of the three shell layers was determined by western blotting. Shell proteins from the nacre, myostracum and fibrous prismatic layer of *M. coruscus* were extracted and divided into two parts, an acid‐soluble (AS) and acid‐insoluble (AIS) fraction, respectively. As shown in Fig. 9A, TLP‐1 was detected with the expected MW in both the AS and AIS matrix fractions obtained from the myostracum layer. In addition, in the AIS fraction from the nacre layer, the band of TLP‐1 weakly appeared.

Using immunohistochemical analysis, the localization of TLP‐1 was also detected in the adductor muscle and mantle. TLP‐1 was expressed mainly inside the muscle fibres and the bottom of the adductor muscle, where it was connected near the shell inner surface (Fig. 9B,C). The expression of rTLP‐1 in the mantle was detected with weak signals inside the middle fold region (Fig. 9D–G).

### Identification of TLP‐1 protein partners in shell matrices

In this study, expressed rTLP‐1 was identified by its His_6_ tag through affinity pull‐down analysis. The partner proteins of rTLP‐1 in the shell matrices were pulled down with a Ni column. After LC‐MS/MS analysis, a set of 17 proteins, including TLP‐1, was identified with FDR < 0.01, and matched more than two unique peptides (Table 1). The MS/MS proteomics data have been deposited to the ProteomeXchange Consortium database (http://proteomecentral.proteomexchange.org) via the iProX partner repository with the data set identifier of IPX0001969004. Most of the partner proteins of rTLP‐1 had been previously identified in the *M. coruscus* shell, such as shell mytilin‐1, collagen‐like protein 2, SD‐rich protein, protease inhibitor‐like protein‐A, calmodulin, actin, whirlin and PDZ domain‐containing protein‐1 [6].

**Table 1:** LC‐MS/MS identification of the protein partners of the rTLP‐1 pulled down from the shell matrices of *Mytilus coruscus* shell.

Protein IDs	Homology protein name (species)	Homologous ID/*E*‐value	Domains (ID) and features	Protein *Q*‐score	Sequence coverage (%)	Unique peptides	Intensity
CL7444.Contig2	Transgelin‐like protein‐1 (*Mytilus coruscus*)	MT240932/0	CH (SM000033)	332	100	18	3.18E+11
CL7857.Contig1	Shell mytilin‐1 (*Mytilus coruscus*)	AKI87978.1/3e‐130	Signal peptide (1–20); Leu (9.9%)	375	100	11	2.25E+11
CL228.Contig1	Tubulin beta chain‐like (*Mizuhopecten yessoensis*)	XP_021372086.1/0.0	Tubulin (SM000864); Tubulin_C (SM000865)	932	100	9	2.00E+09
CL5847.Contig1	SD‐rich protein‐1 (*Mytilus coruscus*)	AKS48139.1/0.0	Internal repeat 1/Ser (17.8%); Gln (10.2%)	686	100	9	3.10E+09
CL4409.Contig1	Collagen‐like protein‐2 (*Mytilus coruscus*)	AKS48142.1/0.0	VWA (SM000327)	280	100	5	8.94E+08
Unigene68573	Whirlin (*Mytilus coruscus*)	QGA67049.1/4e‐83	PDZ (SM000228)	249	100	5	2.07E+10
Unigene28346	ATP synthase subunit beta, mitochondrial‐like (*Pomacea canaliculata*)	XP_025093356.1/0.0	ATP‐synt_ab_N (PF02874); AAA (SM000382)	914	99	4	1.96E+08
CL8938.Contig2	Beta‐actin (*Sus scrofa*)	5NW4_V/0.0	ACTIN (SM000268)	697	100	4	7.03E+09
CL1310.Contig2	PDZ domain‐containing protein‐1 (*Mytilus coruscus*)	AKS48171.1/0.0	Gln (22.5%); Pro (19.4%)	538	100	4	5.13E+08
Unigene66002	Ubiquitin‐like (*Microtus ochrogaster*)	XP_005344417.1/3e‐48	UBQ (SM000213)	205	100	4	6.14E+09
Unigene32537	HSP 90 (*Mytilus coruscus*)	ALL27016.1/0.0	HATPase_c (SM000387); HSP90 (PF00183)	1467	100	4	1.27E+09
CL2091.Contig2	Heat shock protein 70 B2‐like (*Mizuhopecten yessoensis*)	XP_021363224.1/0.0	MreB_Mbl (PF06723)	975	100	2	1.04E+09
Unigene11841	Peptidyl‐prolyl cis‐trans isomerase B‐like (*Crassostrea virginica*)	XP_022330620.1/3e‐70	Pro_isomerase (PF00160)	218	100	2	2.59E+07
CL700.Contig2	Protease inhibitor‐like protein‐A (*Mytilus coruscus*)	AKS48173.1/2e‐96	Signal peptide (1–22); NTR (SM000206)	283	89	2	3.00E+08
Unigene58608	Collagen‐like protein‐1 (*Mytilus coruscus*)	AKS48145.1/2e‐21	VWA (SM000327)	89.7	100	2	5.12E+08
Unigene33253	14‐3‐3 protein epsilon isoform X2 (*Chanos chanos*)	XP_030634506.1/2e‐151	14_3_3 (SM000101)	431	100	2	2.12E+08
CL3579.Contig2	Calmodulin‐A‐like isoform X2 (*Pomacea canaliculata*)	XP_025100478.1/5e‐73	3*EFh (SM000054)	293	100	2	4.88E+08

**Fig. 10:**
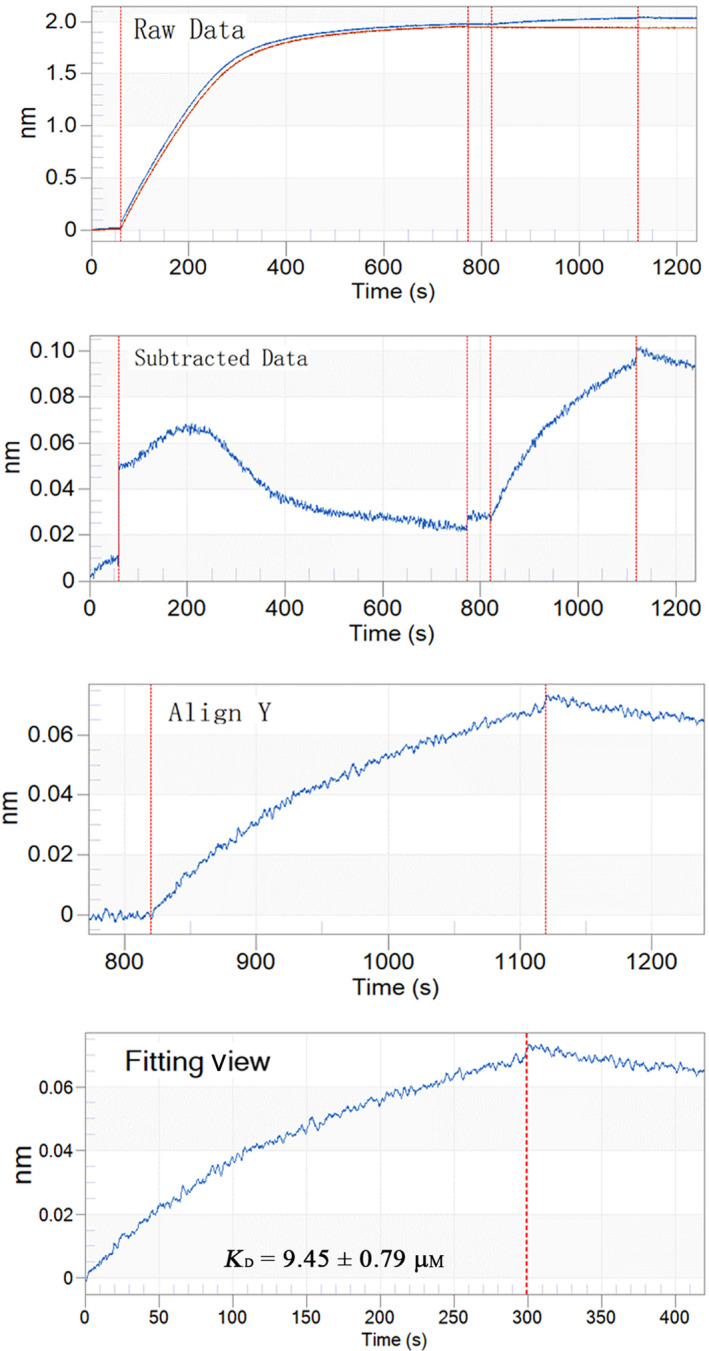
BLI curves (from the raw data to the final fitting view) for the binding of rTLP‐1 to biosensors coated with actin.

Considering the abundant actins identified in the shell of *Mytilus* [6, 16] and the possible roles of TLP‐1 in the attachment of the adductor muscle shell, the binding of rTLP‐1 with actin was measured by BLI. The data are summarized in Fig. 10. The *K*_D_ of rTLP‐1 with actin was calculated by fortebio data analysis software (Sartorius BioAnalytical Instruments, Inc., Bohemia, NY, USA) as 9.45 ± 0.79 μm (*n* = 3), indicating a strong interaction between rTLP‐1 and actin.

**Fig. 11:**
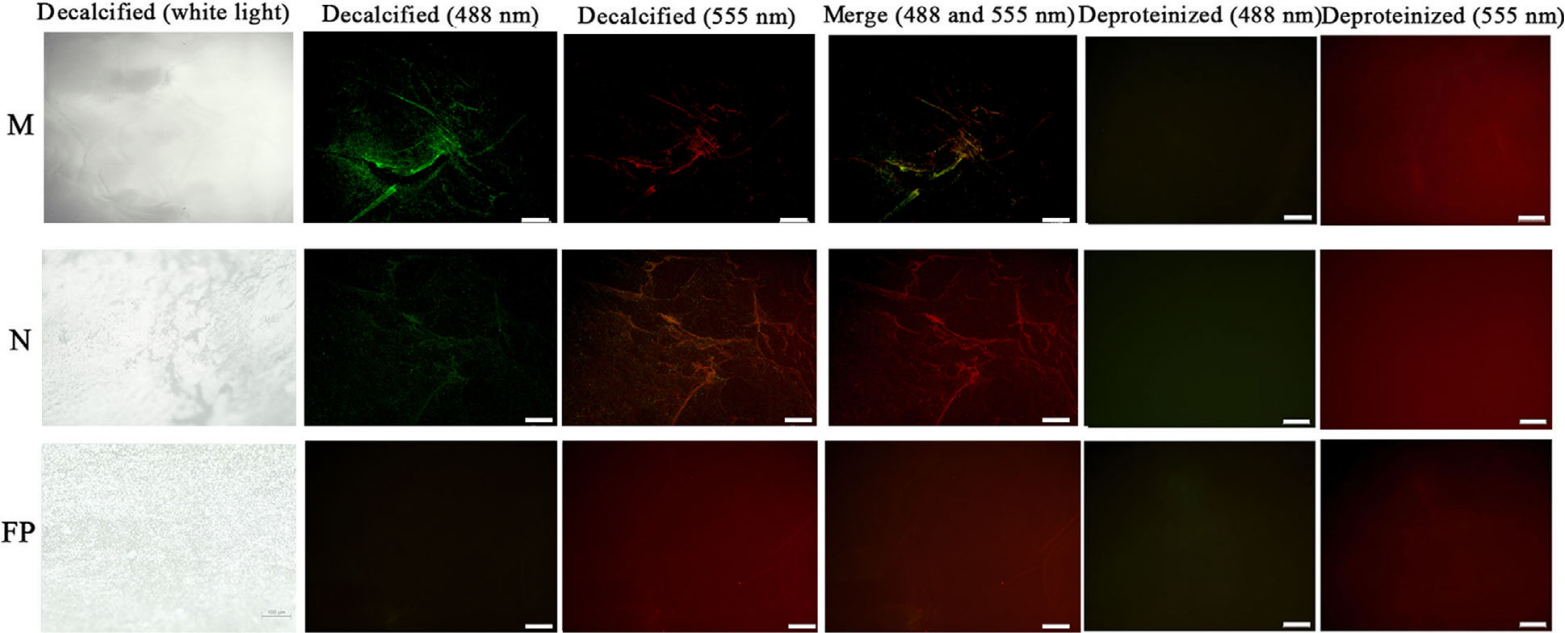
Immunofluorescence location of native TLP‐1 (green at 488 nm) and actin (red at 555 nm) on the surface of decalcified shell samples of *Mytilus coruscus*, and the deproteinized shell samples were used as negative controls. N, nacre; M, myostracum; FP, fibrous prism. The scale bar, 100 µm.

The localization of rTLP‐1 with actin in the shell inner surface was also detected by double‐labelling immunofluorescence. As shown in Fig. 11, organic membranes with different textures were on the inner shell myostracum, nacre and fibrous prism layers after decalcification. Both rTLP‐1 (green) and actin (red) signals were observed in the myostracum and nacre layers of the inner surface of the decalcified shell. Most of the rTLP‐1 and the actin signals were observed in the same region, suggesting an interaction between these two proteins (Fig. 11). No rTLP‐1 signal was detected in the fibrous prismatic layer, but a weak signal of actin was observed in this layer. For the deproteinated shell samples, no rTLP‐1 or actin signal was detected (Fig. 11).

## Discussion

Shell matrix proteins with CH domains, such as TLP, calponin and filamin [6, 16, 17, 18], have been previously identified in various Mollusca shells, indicating possible biomineralization‐related functions of the CH domain in shell formation. The CH domain has been found in cytoskeletal and signal transduction proteins, including actin‐binding proteins, such as spectrin, β‐actinin, dystrophin, utrophin and fimbrin, which are essential for regulating cell shape and signalling [22]. Transgelin, first identified in 1987 [8], is a member of the calponin family and a CH domain‐containing protein with multiple functions involved in regulating muscle development [10], muscle contraction [11, 12], cell migration [23] and tissue remodelling [24] in vertebrates. Functional studies of transgelin in invertebrates, especially in the shell formation of molluscs, are rare. However, calponin, a CH domain‐containing protein, has been reported as a factor during the process of bone mineralization in vertebrates [20, 25]. In addition, calponin in *C. farreri* was recently reported to function in the shell regeneration process after shell notching, indicating a biomineralization‐related role for calponin during shell formation [21]. These results indicated a possible role of the CH domain in biomineralization.

TLP‐1 is a basic protein with a theoretical molecular mass of 18.9 kDa and a *pI* of 8.28. Lys is the most abundant amino acid in its sequence (Table S2). Although acidic SMPs have been reported as key macromolecules for shell formation with the Asp or Glu in the SMPs suggested as possible Ca^2+^‐binding residues [26, 27], basic SMPs with abundant Lys/Arg have also been reported to play important roles in biomineralization [28, 29, 30]. For TLP‐1, most Lys residues are presented as ‘–KK‐’ dipeptide fragments in the sequence (Fig. 1), and we speculated that the basic Lys‐rich region of TLP‐1 may interact with acidic SMPs or CO_3_^2−^ and be involved in protein cross‐linking and crystallization. In the secondary structure of TLP‐1 (Fig. 2B), α‐helix is the predominant conformation, with six a‐helices forming a compact conformation containing a potential EF‐hand motif, which was also found in other transgelin structures [31], indicating the possibility of transgelin binding to Ca^2+^. The blast search results revealed a high sequence identity (36–92%) of TLP‐1 with CH domain‐containing proteins (including transgelin, calponin and myophilin) in other invertebrates, and the phylogenetic analysis revealed that the clade of TLP‐1 (*Mytilus*) was grouped with *Crassostrea* and *Mizuhopecten*, indicating the conservation of this protein among shell‐forming *bivalves* (Fig. 3).

In the current work, TLP‐1 of *M. coruscus* was successfully expressed using a prokaryotic recombinant expression system based on a codon optimization strategy. The effects of rTLP‐1 on CaCO_3_ crystal morphology and polymorph formation, crystallization rate and binding ability with CaCO_3_ crystals were examined. The results showed that rTLP‐1 induced morphological changes in aragonite (Fig. 7) and polymorphic changes in calcite (Fig. 6), exhibited binding with calcite (Fig. 8A), and educed the crystallization rate of calcite (Fig. 8B). The effects of rTLP‐1 on CaCO_3_ crystallization can be partially explained by previous findings showing that SMPs have the ability to bind to the surface of CaCO_3_ crystals, reducing the growth of crystals accordingly, and ultimately promote crystal polymorph formation with different morphologies [32]. Interestingly, rTLP‐1 showed relatively stronger effects on calcite than aragonite, a finding similar to the effects of two other shell proteins, whirlin‐like protein (WLP) [33] and collagen‐like protein 2 (CLP‐2) [34]. Both WLP and CLP‐2 are myostracum‐related shell proteins with selective action for calcite. Considering that both the myostracum and the nacre have aragonite with different morphologies, it is possible that TLP‐1, identified exclusively in the myostracum layer [6], has important functions in the formation of the myostracum layer, such as the transformation of calcite to aragonite, retention of aragonite in the myostracum and morphological change induction in the aragonite crystals of the myostracum.

The expression level of TLP‐1 in various tissues was determined by qPCR, and the results showed that TLP‐1 showed the highest expression level in the adductor muscle (Fig 4A). Although the mantle was previously reported as the main tissue in which most SMPs were expressed, some SMP genes were found to be expressed in nonmantel tissues [35, 36], suggesting that these SMPs may have multiple functions. Transgelin is a protein family with multiple functions mediated by its CH domain [14]. The high expression level of TLP‐1 in adductor muscle and blood cells suggests that it may have fundamental roles in addition to shell formation, such as muscle contraction [11, 12] and/or immune functions [37]. Because the mantle is the main tissue that undergoes biomineralization, *in situ* hybridization assays of TLP‐1 in the mantle were performed, and the localization of the TLP‐1 gene was observed in the epithelial cells of the middle fold and outer fold (Fig. 4E), suggesting a function of TLP‐1 in the formation of the prismatic layer [38, 39]. Immunohistochemical analysis by anti‐rTLP‐1 antibody further confirmed the localization of TLP‐1 in the middle fold of the mantle (Fig. 9D–G). The signal of TLP‐1 in the mantle detected by immunohistochemistry was weaker than that shown by *in situ* hybridization, suggesting a low abundance of native TLP‐1 in the mantle. We speculate that most of the expressed TLP‐1 may be transported from the mantle to the shell via the vesicular trafficking system, a principal pathway for the transportation of SMPs without signal peptides [35]. In addition, the strong TLP‐1 signal at the bottom of the adductor muscle, detected by both *in situ* hybridization (Fig. 4C) and immunohistochemistry (Fig. 9C), suggested the possible function of TLP‐1 in muscle‐shell attachment, because the adductor muscle is connected with the myostracum layer via organic membranes at the bottom of the adductor muscle and on the surface of the myostracum layer [6].

As emphasized previously, the assembly of a molecular framework is necessary for shell formation [40]. In the current study, a set of candidate proteins that interact with TLP‐1 was identified by pull‐down techniques combined with LC‐MS/MS analyses (Table 1). The 17 protein partners of TLP‐1 can be divided into three categories according to their localizations and functions, including cytoskeleton‐related proteins (tubulin, collagen and actin), biomineralization‐related proteins (shell mytilin‐1, 14‐3‐3 protein, PDZ protein, calmodulin, protease inhibitor‐like protein and SD‐rich protein) and others (HSPs, ubiquitin, etc.) (Table 1). Most of the pulled‐down proteins, such as collagen and biomineralization‐related shell proteins, were identified in the shell proteome of *M. coruscus* [6], indicating a possible protein network in the shell mediated by TLP‐1. Among the identified TLP‐1 protein partners, actin was identified in the shell of *M. coruscus* [6] and has been reported as a partner of transgelin [41]. Therefore, BLI was used to test the affinity of actin with TLP‐1, and the results definitively showed the binding ability of rTLP‐1 with actin (Fig. 10). Double‐labelling immunofluorescence was also performed to determine the localization of TLP‐1 together with actin. The results showed substantial signals of the two proteins and an overlapping pattern of TLP‐1 and actin on the shell inner surface in the myostracum layer (Fig. 11). Although the identification of cytoskeletal proteins, such as actin, in various mollusc shells has been controversial [42], we cannot exclude the possibility that actin may participate in biomineralization by interacting with actin‐binding proteins (such as TLP‐1 in the present study) and forming a framework during shell formation. Our data provide clues for exploring the protein–protein interactions of SMPs and for understanding the supramolecular chemistry that contributes to shell formation. However, more detailed studies are necessary to explore the real interaction between TLP‐1 and the identified protein partners.

## Materials and methods

### Sequence analysis and the expression of the TLP‐1 gene in *M. coruscus* tissues

The full‐length cDNA sequence of TLP‐1 was screened from the transcriptomic data of the *M. coruscus* mantle and verified by LC‐MS/MS analysis [6]. The cDNA sequence of TLP‐1 was confirmed using PCR with primers 5′‐AAAGCGGTCTTGGGTATA‐3′ (forward primer) and 5′‐GGTGACTTTCCATCTTTTTTC‐3′ (reverse primer) and verified by PCR sequencing. The sequence of TLP‐1 was analysed using conventional bioinformatics tools, including ORF Finder, blast and SignalP servers; mega 7 software (Center for Evolutionary Medicine and Informatics, The Biodesign Institute, Tempe, AZ, USA); and the SMART domain prediction, Phyre secondary structure prediction, and SWISS‐MODEL tertiary structure prediction tools.

According to the protocol of a previous study [33], quantitative real‐time PCR (qRT‐PCR) was used to evaluate the expression level of TLP‐1 in various tissues. Briefly, total RNA was extracted from various tissues (mantle, adductor muscle, foot, gill, blood and gonad) by TRIzol reagent. The first strand of cDNA was synthesized using the PrimeScript™ RT kit (TaKaRa Bio Inc., Kusatsu, Shiga, Japan). The qRT‐PCR analyses were performed with three independent replicates using SYBR^®^ Premix Ex Taq™ (TaKaRa) on an MX3000P Real‐Time PCR system (Stratagene, La Jolla, CA, USA). Specific primers derived from the TLP‐1 sequence were used for qRT‐PCR, including TLP‐1/F (5′‐AAAGCGGTCTTGGGTATA‐3′) and TLP‐1/R (5′‐GGTGACTTTCCATCTTTTTTC‐3′). The relative expression levels were measured using the 2^−ΔΔCt^ method [43] with β‐actin as an internal reference (β‐actin/F: 5′‐ATGAAACCACCTACAACAGT‐3′; β‐actin/R: 5′‐TAGACCCACCAATCCAGACG‐3′). The cycling conditions were as follows: 3 min at 94 °C for 40 cycles of 94 °C for 10 s, 60 °C for 40 s, 72 °C for 5 s and 72 °C for 30 s. The statistical analysis of differences was performed by SPSS 16.0 with one‐way ANOVA followed by Tukey's multiple range test. Differences were considered significant at *P* < 0.05.

### *In situ* hybridization of TLP‐1

The localization of TLP‐1 mRNA expressed in the mantle and adductor muscle was determined by *in situ* hybridization using a previously reported protocol [33, 34]. The fixed tissues were dehydrated through an ethanol series and then subjected to a xylene bath prior to paraffin embedding. Paraffin blocks were sliced into 5‐μm‐thick sections. After treatment with proteinase K at 37 °C for 20 min, the sectioned tissues were washed with 0.1 m freshly prepared glycine solution for 1 min and PBS for 2 min, respectively. The tissues were immediately fixed in 4% paraformaldehyde for 10 min, followed by incubation at 65 °C with a FAM‐labelled probe (5′‐FAM‐ACCAUUGAUAGCAGUAAGCACAUCUGUC‐3′) for 48 h. The sectioned tissues were washed in formamide‐4 × SSC at 60 °C. The signals of the control samples were visualized with a substrate 4′, 6‐diamidino‐2‐phenylindole (DAPI) reagent, and the positive signals were visualized at an excitation wavelength of 492 nm and an emission wavelength of 518 nm.

### Recombinant expression and purification of TLP‐1

The TLP‐1 gene coding the mature peptide was recombinantly expressed in an *E. coli* expression system after codon optimization. A *pET/28α* expression vector was constructed and designed to yield a recombinant protein product with an MW of ~ 19 kDa (with a His_6_ tag). The recombinant TLP‐1 was expressed in *E. coli* strain BL21 (DE3) according to the procedure of previous work [33]. *E. coli* cells containing *rTLP‐1/pET28α* were grown at 37 °C in Luria–Bertani liquid medium (Sangon Biotech, Shanghai, China) with 10 μg·mL^−1^ ampicillin. The cells were treated with IPTG (1 mm) for gene induction and grown for 4 h. After gene induction, the cells were harvested and centrifuged at 1500 ***g*** for 15 min at 4 °C, and the pellets were dissolved in ice‐cold lysis buffer and homogenized using a sonicator at 4 °C. The inclusion bodies were harvested by centrifugation (8000 ***g***, 10 min, 4 °C), dissolved in PBS buffer containing 8 m urea and 10 mm imidazole (pH 8.0) and purified by elution buffer containing 8 m urea and 200 mm imidazole (pH 8.0) on a Ni‐NTA column. Isolated rTLP‐1 was refolded in a buffer containing oxidized and reduced glutathione and dialysed in a graded concentration of urea (0–8 m) according to the protocol in [44]. Refolded rTLP‐1 was further purified on a reversed‐phase C8 column (4.6 mm × 250 mm, 300 Å; Agilent Technologies Inc., Chandler, USA) by high‐performance liquid chromatography (HPLC, Waters 650E, Milford, MA, USA). The eluted protein fraction from the HPLC column was collected, lyophilized, and stored at −20 °C before use. SDS/PAGE with a 12% polyacrylamide gel was performed, and the protein bands were visualized using Coomassie Brilliant Blue G‐250.

### Functional analysis of rTLP‐1

The effects of rTLP‐1 on the morphology of CaCO_3_ crystals (calcite and aragonite) were tested by *in vitro* crystal growth experiments [33, 45]. Briefly, rTLP‐1 was incubated with a freshly prepared saturated solution of calcium carbonate [46] on a siliconized cover glass with MgCl_2_ (for the preparation of aragonite) or without MgCl_2_ (for the preparation of calcite). Crystallization experiments were carried out with 10, 30 and 50 μg·mL^−1^ rTLP‐1. The morphologies and polymorphs of the CaCO_3_ crystals were observed by a Nova Nano 450 (FEI) scanning electron microscope (SEM) and FTIR spectroscopy (Nicolet Nexus 670, Thermo Nicolet Corporation, Madison, WI, USA), respectively.

The interaction between rTLP‐1 and CaCO_3_ crystals was tested by SDS/PAGE according to a previously reported protocol [33, 46]. Briefly, 1 mg·mL^−1^ rTLP‐1 was dissolved in water (sample I) and then incubated with calcite or aragonite crystals at room temperature for 2 h. After centrifugation (10 000 ***g*** for 15 min), the supernatants were collected as sample II. The sediments were decalcified and centrifuged (10 000 ***g*** for 15 min), and the supernatants were dialysed (with a 1 kDa cut‐off) and used as sample III. Samples I–III were analysed by SDS/PAGE.

The inhibition calcium carbonate precipitation by rTLP‐1 was tested using a previously reported protocol [33, 45]. Briefly, 10 mm CaCl_2_ containing rTLP‐1 at various concentrations (10, 30, and 50 μg·mL^−1^) was dropped onto a 96‐well plate, and the plate was placed in a closed desiccator. Solid ammonium carbonate was added to the desiccator for calcium carbonate precipitation. The turbidity of the calcium chloride solution was monitored every minute for 10 min by measuring the absorbance at 630 nm with a microplate reader (Synergy H1; BioTek, Winooski, VT, USA).

### Polyclonal antibody preparation and immunohistochemical analysis

Purified rTLP‐1 was enriched and submitted to HuaAn Biotechnology Co., Ltd. (Hangzhou, China) for the production of polyclonal antibodies by immunizing New Zealand rabbits. The antiserum was collected and purified with a protein A/G column. The specificity of the antibodies was assayed by western blotting with acid‐soluble and acid‐insoluble matrices extracted from three shell layers (nacre, myostracum and fibrous prism) of *M. coruscus*. The shell matrices were extracted as described previously [6, 33], and the matrix proteins were separated by SDS/PAGE. The PAGE gel was then transblotted onto a PVDF membrane. The anti‐rTLP‐1 polyclonal antibody (1 : 2000) was used as the primary antibody, and a horseradish peroxidase‐labelled goat anti‐rabbit IgG (1 : 10 000; HuaAn Biotechnology Co., Ltd.) was used as the secondary antibody. Blots were visualized using a 3,3′,5,5′‐tetramethylbenzidine‐stabilized substrate.

To determine the localization of natural TLP‐1, the mantle and adductor muscle of *M. coruscus* were collected and fixed in 10% formaldehyde overnight and then dehydrated through an ascending ethanol gradient. Sections (4 μm) were cut by a microtome and collected on coated slides for immunohistochemical analysis. After dewaxing and rehydration, the slides were incubated overnight with the anti‐rTLP‐1 antibody (1 : 200) supplemented with 1% BSA at 37 °C. The primary antibodies were detected using a peroxidase‐conjugated antibody against rabbit IgG and stained with a diaminobenzidine (DAB) solution. The sections were examined and photographed using a microscope (DFC450C; Leica, Wetzlar, Germany).

### His‐tag affinity pull‐down assay

A His‐tag affinity pull‐down assay was performed to identify the protein partners of TLP‐1 in the shell matrices of *M. coruscus* using the protocol of Jiang *et al*. [33]. Ni‐NTA beads (Sangon, Shanghai, China) were used for binding rTLP‐1 at the His_6_ tag contained in the sequence, and the beads were washed with binding buffer (20 mm Tris/HCl, 150 mm NaCl and 10 mm imidazole at pH 8.0) and then incubated for 4 h with total proteins extracted from the shell matrices of *M. coruscus* at 4 °C and then washed with elution buffer (20 mm Tris/HCl, 300 mm NaCl and 300 mm imidazole at pH 8.0). The eluted fractions were analysed by LC‐MS/MS after digestion by trypsin. The LC‐MS/MS experiments were performed on a Q‐Exactive Plus MS spectrometer coupled with an Easy‐nLC chromatograph (Thermo Scientific, Waltham, Massachusetts, USA). The MS data were analysed using maxquant software (version 1.6.1.0., Max‐Planck Institute of Biochemistry, Netherlands, Germany) and searched against the mantle transcriptome database of *M. coruscus* (Accession: SRX792025) [6]. The database search results were filtered and exported based on a < 1% false discovery rate (FDR) at the peptide‐spectrum‐matched level and protein level.

### Biolayer interferometry

The binding of rTLP‐1 with actin was measured by biolayer interferometry (BLI) on an Octet RED BLI (Pall ForteBio) at 25 °C, according to a previous protocol [33, 47]. Briefly, rTLP‐1 (1 mm) was dissolved in PBS buffer (pH 7.4) containing 0.05% (v/v) Tween 20 and 0.1% (v/v) BSA and then loaded onto an APS biosensor (Pall ForteBio) coated with actin and incubated in PBS buffer (pH 7.4). The procedure was as follows: 60 s for baseline 1, 900 s for loading, 300 s for baseline 2, 300 s for association and 120 s for dissociation. The raw data were processed by subtraction and alignment, and the affinity constant (*K*_D_) was determined using fortebio data analysis 10.0 software [48].

### Double‐labelling immunofluorescence analysis

The location of rTLP‐1 together with actin on the shell inner surface was determined by double‐labelling immunofluorescence according to a previous protocol [33]. Two antibodies were used for the detection of rTLP‐1 and actin, including a rabbit anti‐rTLP‐1 antibody (1 : 500, prepared for this study) and mouse anti‐actin monoclonal antibody (1 : 500, Hangzhou HuaAn). The shell of the adductor muscle scar region was cut into pieces of ~ 1 cm^2^, and the shell pieces were washed and sonicated in 5% NaOH to remove the remaining adductor muscle tissue and other organic contaminants. Then, the shell pieces were soaked for 24 h in a stationary liquid containing 10% formaldehyde and 4% formic acid. The shell pieces were further treated for 30 min with 0.25% Triton X‐100 and blocked for 1 h with 10% negative goat serum at 37 °C. Immunostaining was performed by incubating the shell samples with rabbit anti‐rTLP‐1 polyclonal antibody (1 : 500) and Alexa Fluor 488‐conjugated goat anti‐rabbit antibody (1 : 5000). The stained sections were examined with a fluorescence microscope (DMIL LED FLUO; Leica) equipped with a DFC450C digital imaging system (Leica). Deproteinized shell samples treated for 1 h with 20% NaOH at 60 °C were used as negative controls. The same protocol was used for detecting the actin with mouse anti‐actin monoclonal antibody and Alexa Fluor 555‐conjugated goat anti‐mouse antibody (1 : 5000).

## Conflict of interest

The authors declare no conflict of interest.

## Author contributions

YJ planned and performed experiments; analysed data; discussed results; and wrote paper. QS performed experiments and analysed data. MF analysed data. JH supported experiments and analysed data. XZ supported experiments and discussed results. HX supported experiments and discussed results. ZL planned experiments; analysed data; discussed results; and wrote paper.

## Supporting information


**Fig. S1.** Multiple sequence alignment of TLP‐1 with homologues retrieved from NCBI nr database searching.

**Table S1.** The accession No., protein name, and species of homologues of TLP‐1.

**Table S2.** Amino acid composition (mole percent) of TLP‐1.

## Data Availability

Datasets are in a publicly accessible repository: the sequence of TLP‐1 is available in GenBank with Accession of MT240932; The MS/MS proteomics data of partners binding with TLP‐1 have been deposited to the ProteomeXchange Consortium database (http://proteomecentral.proteomexchange.org) via the iProX partner repository with the data set identifier of IPX0001969004. Other data will be available from the corresponding author upon reasonable request.
