# Supplementary figures and images for: Recombinant transgelin‐like protein 1 from Mytilus shell induces formation of CaCO3 polymorphic crystals in vitro
Source: FEBS Open Bio. 2020 Sep 21;10(10):2216–34. doi: 10.1002/2211-5463.12972 (PMC7530383; doi:10.1002/2211-5463.12972)

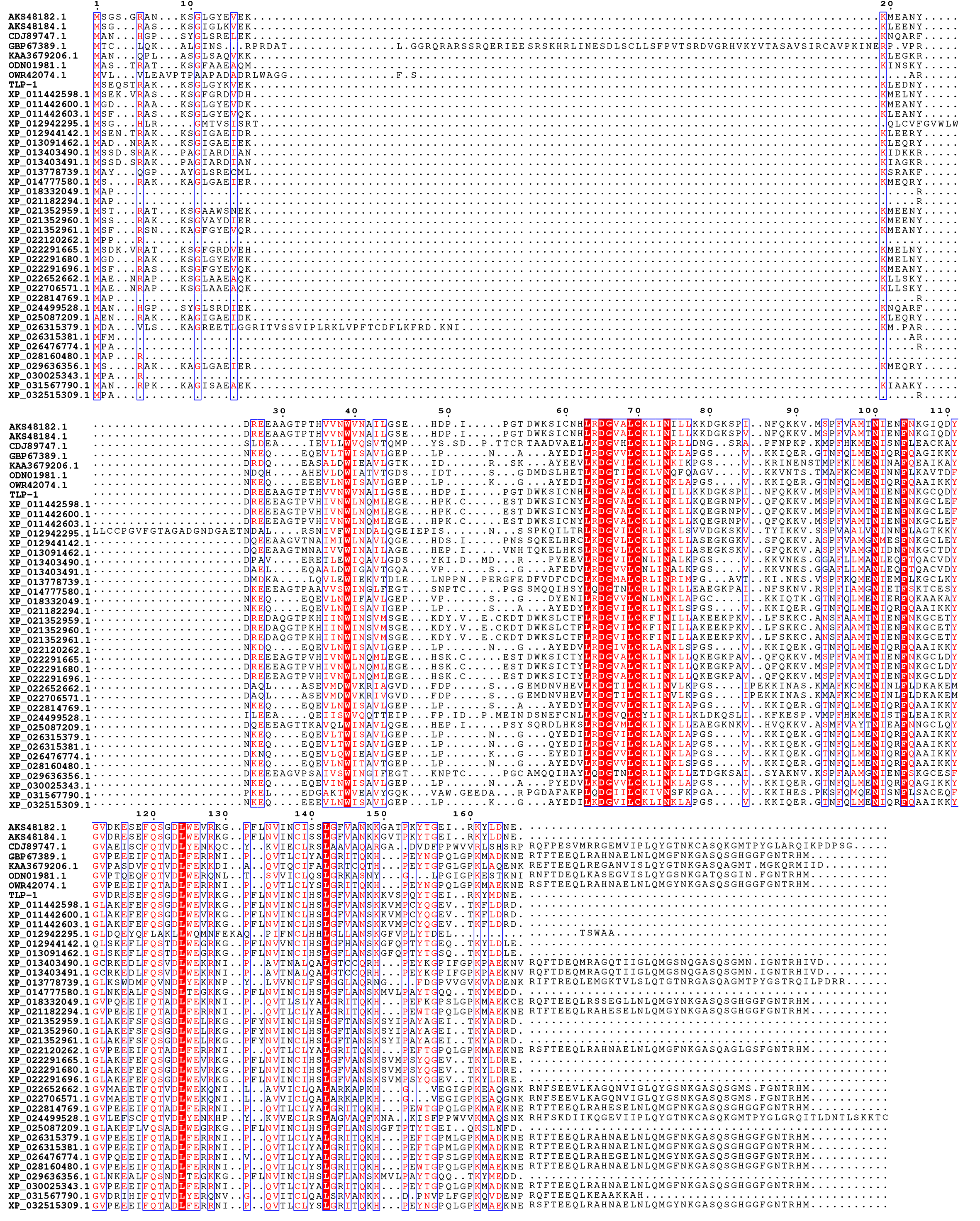

Supplement: Supplementary file 1 — Fig. S1. Multiple sequence alignment of TLP‐1 with homologues retrieved from NCBI nr database searching. [file FEB4-10-2216-s001.tif]
