# Supplementary material for: Recombinant transgelin‐like protein 1 from Mytilus shell induces formation of CaCO3 polymorphic crystals in vitro
Source: FEBS Open Bio. 2020 Sep 21;10(10):2216–34. doi: 10.1002/2211-5463.12972 (PMC7530383; doi:10.1002/2211-5463.12972)
Supplement: Supplementary file 2 — Table S1. The accession No., protein name, and species of homologues of TLP‐1. [file FEB4-10-2216-s002.docx]

**Supplementary Table 1**

| **Accession No. and protein name** | **Species** |
| --- | --- |
| MT240932 TLP-1 | *Mytilus coruscus* |
| AKS48184.1 transgelin-like protein-1 | *Mytilus galloprovincialis* |
| AKS48182.1 transgelin-like protein-2 | *Mytilus galloprovincialis* |
| XP_022291680.1 transgelin-3-like isoform X2 | *Crassostrea virginica* |
| XP_022291696.1 rac guanine nucleotide exchange factor B-like isoform X3 | *Crassostrea virginica* |
| XP_011442603.1 transgelin-3 isoform X3 | *Crassostrea gigas* |
| XP_011442600.1 transgelin-3 isoform X2 | *Crassostrea gigas* |
| XP_022291665.1 transgelin-2-like isoform X1 | *Crassostrea virginica* |
| XP_011442598.1 transgelin-3 isoform X1 | *Crassostrea gigas* |
| XP_021352960.1 transgelin-3-like isoform X2 | *Mizuhopecten yessoensis* |
| XP_021352959.1 transgelin-3-like isoform X1 | *Mizuhopecten yessoensis* |
| XP_021352961.1 transgelin-3-like isoform X3 | *Mizuhopecten yessoensis* |
| XP_013091462.1 transgelin-3-like | *Biomphalaria glabrata* |
| XP_012944142.1 rac guanine nucleotide exchange factor B-like | *Aplysia californica* |
| XP_025087209.1 transgelin-2-like | *Pomacea canaliculata* |
| XP_014777580.1 transgelin-3-like | *Octopus bimaculoides* |
| XP_029636356.1 transgelin-3-like | *Octopus vulgaris* |
| XP_012942295.1 transgelin-like | *Aplysia californica* |
| XP_024499528.1 calponin | *Strongyloides ratti* |
| CDJ89747.1 Calponin | *Haemonchus contortus* |
| XP_030025343.1 myophilin | *Manduca sexta* |
| XP_031567790.1 myophilin-like isoform X1 | *Actinia tenebrosa* |
| XP_013403491.1 muscle-specific protein 20 isoform X2 | *Lingula anatina* |
| XP_013403490.1 myophilin isoform X1 | *Lingula anatina* |
| GBP67389.1 Myophilin | *Eumeta japonica* |
| XP_022706571.1 myophilin-like | *Varroa jacobsoni* |
| XP_022814769.1 myophilin | *Spodoptera litura* |
| XP_013778739.1 myophilin-like | *Limulus polyphemus* |
| XP_032515309.1 myophilin | *Danaus plexippus plexippus* |
| XP_026476774.1 myophilin | *Ctenocephalides felis* |
| XP_022652662.1 myophilin-like | *Varroa destructor* |
| ODN01981.1 Myophilin | *Orchesella cincta* |
| KAA3679206.1 transgelin | *Paragonimus westermani* |
| OWR42074.1 calponin/transgelin | *Danaus plexippus plexippus* |
| XP_021182294.1 myophilin | *Helicoverpa armigera* |
| XP_022120262.1 myophilin | *Pieris rapae* |
| XP_018332049.1 muscle-specific protein 20 | *Agrilus planipennis* |
| XP_026315381.1 myophilin isoform X3 | *Hyposmocoma kahamanoa* |
| XP_028160480.1 myophilin | *Ostrinia furnacalis* |
| XP_026315379.1 muscle-specific protein 20 isoform X1 | *Hyposmocoma kahamanoa* |
