# Supplementary material for: Recombinant transgelin‐like protein 1 from Mytilus shell induces formation of CaCO3 polymorphic crystals in vitro
Source: FEBS Open Bio. 2020 Sep 21;10(10):2216–34. doi: 10.1002/2211-5463.12972 (PMC7530383; doi:10.1002/2211-5463.12972)
Supplement: Supplementary file 3 — Table S2. Amino acid composition (mole percent) of TLP‐1. [file FEB4-10-2216-s003.docx]

**Supplementary Table 2**

| Amino acid | Mole percent |
| --- | --- |
| Ala (A) | 4.2% |
| Arg (R) | 3.6% |
| Asn (N) | 7.8% |
| Asp (D) | 6.0% |
| Cys (C) | 2.4% |
| Gln (Q) | 3.0% |
| Glu (E) | 7.2% |
| Gly (G) | 7.8% |
| His (H) | 2.4% |
| Ile (I) | 6.0% |
| Leu (L) | 6.6% |
| Lys (K) | 10.8% |
| Met (M) | 2.4% |
| Phe (F) | 3.6% |
| Pro (P) | 4.2% |
| Ser (S) | 6.6% |
| Thr (T) | 3.6% |
| Trp (W) | 1.8% |
| Tyr (Y) | 3.0% |
| Val (V) | 7.2% |
| Pyl (O) | 0.0% |
| Sec (U) | 0.0% |
